# Supplementary material for: The Sink-Source Relationship in Cucumber (Cucumis sativus L.) Is Modulated by DNA Methylation
Source: Plants (Basel). 2023 Dec 28;13(1):103. doi: 10.3390/plants13010103 (PMC10780960; doi:10.3390/plants13010103)
Supplement: Supplementary file 1 [file plants-13-00103-s001.zip › supplemental figure.pdf]

plants

MDPI

Article

The Sink–Source Relationship in Cucumber (*Cucumis sativus* L.) is Modulated by DNA Methylation

Yudan Wang [1], Huimin Zhang [2], Jiawen Gu [1], Chen Chen [1], Jiexia Liu [1], Zhiping Zhang [1], Bing Hua [1], Minmin Miao [1,3,4,*]

[1] College of Horticulture and Landscape Architecture, Yangzhou University, Yangzhou 225009, China; DX120210135@stu.yzu.edu.cn (Y.W.); 394256434@qq.com (H.Z.); MZ120211347@stu.yzu.edu.cn (J.G.); kukujujubo222@163.com (C.C.); 008302@yzu.edu.cn (J.L.); zhangzp@yzu.edu.cn (Z.Z.); Binghua@yzu.edu.cn (B.H.)
[2] Jiangsu Yanjiang Institute of Agricultural Sciences, Nantong 226541, China
[3] Joint International Research Laboratory of Agriculture and Agri-Product Safety of Ministry of Education of China, Yangzhou University, Yangzhou 225009, China
[4] Key Laboratory of Plant Functional Genomics of the Ministry of Education/Jiangsu Key Laboratory of Crop Genomics and Molecular Breeding, Yangzhou University, Yangzhou 225009, China
* Correspondence: mmmiao@yzu.edu.cn

Supplementary Figures

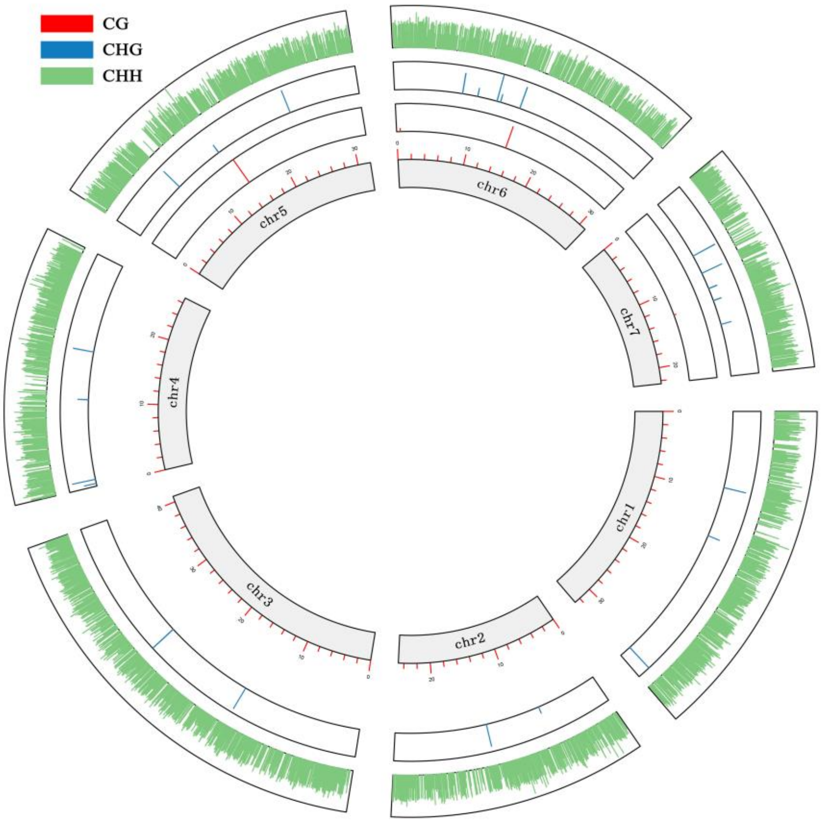

Figure S1. The location of DMRs on the genome.

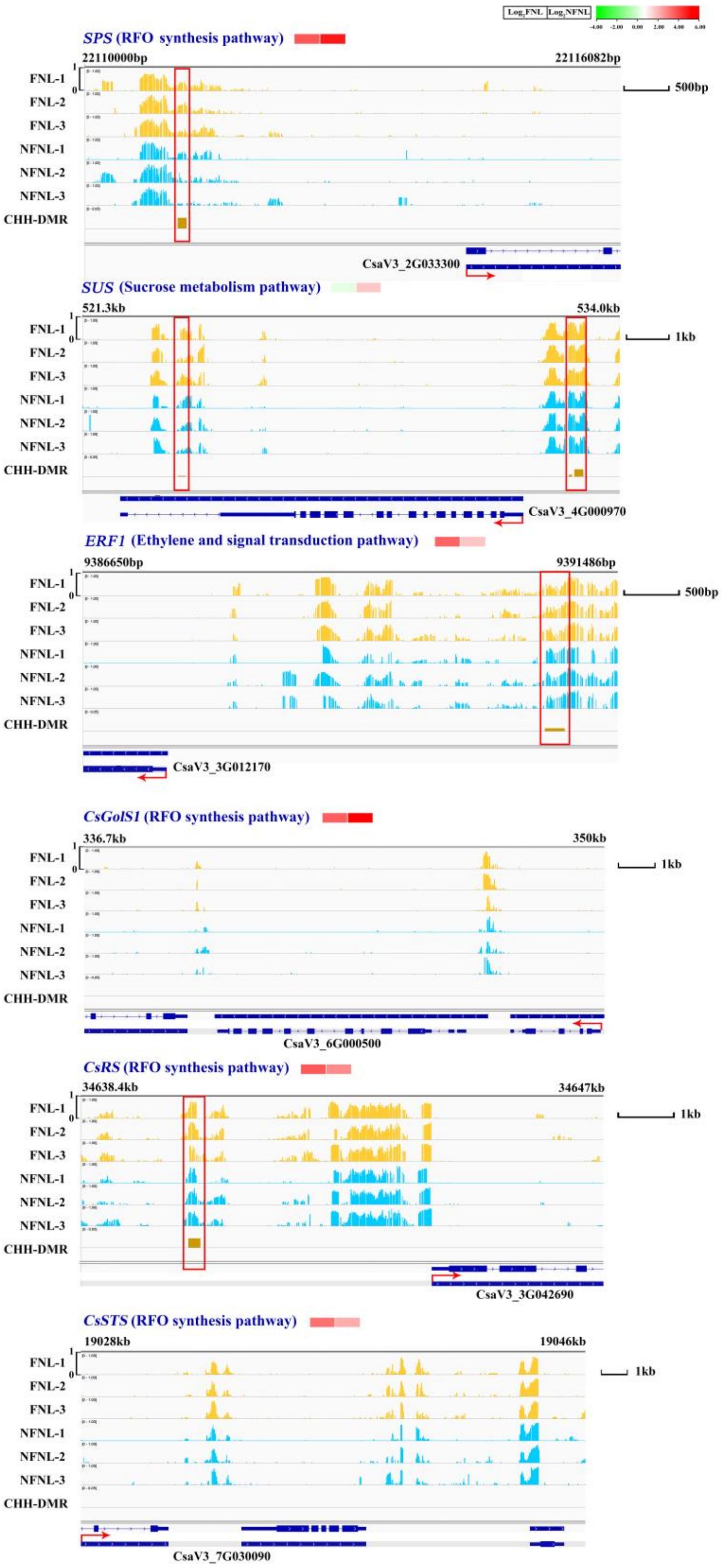

Figure S2. Methylation level and differentially methylated regions (DMRs) of vital genes in the metabolism pathways related to the sink-source regulation**.**

The areas marked by the brown boxes are the DMRs, Higher brown bars represent greater differences in DNA methylation levels between FNLs and NFNLs. Yellow represents FNL, blue represents NFNL, and higher yellow and blue bars represent higher levels of DNA methylation at that location. Red arrows represent the direction of transcription.

Heatmap represents the expression levels of differentially expressed genes (DEGs) in NFNLs and FNLs in RNA-seq. Log_2 (FNLs/NFNLs) represents the gene fold-change, where FNLs/NFNLs refers to the ratio of FPKM reads.

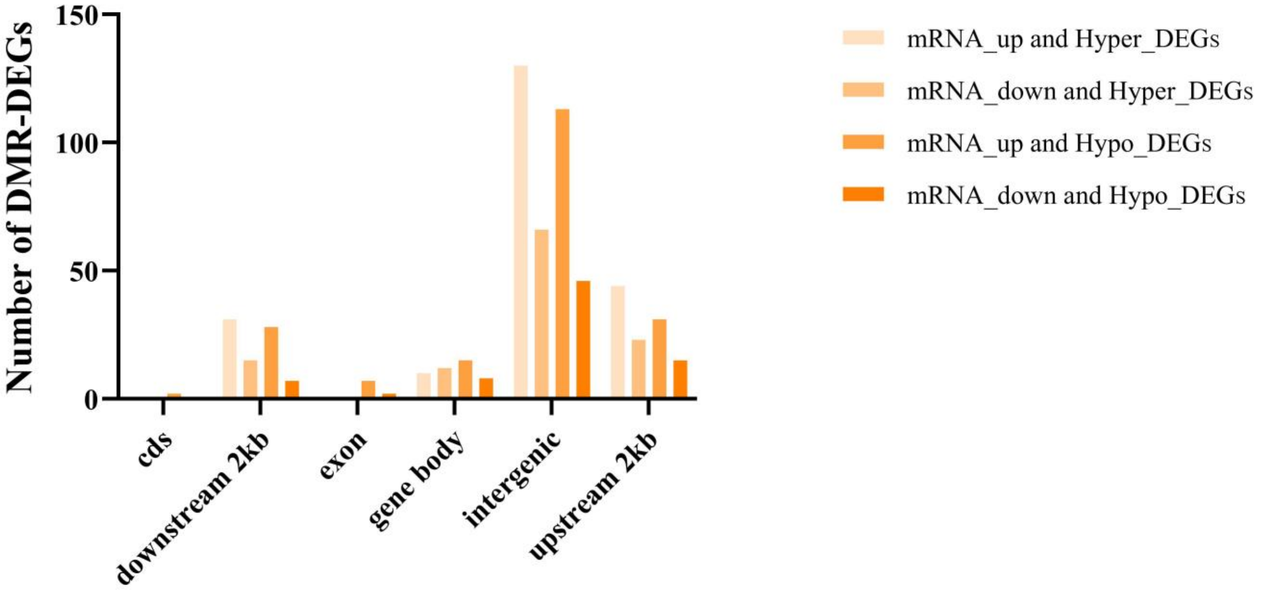

Figure S3. DMR-DEGs in different gene elements.
